# Supplementary material for: The causal relationship between the human gut microbiota and pyogenic arthritis: a Mendelian randomization study
Source: Front Cell Infect Microbiol. 2024 Nov 26;14:1452480. doi: 10.3389/fcimb.2024.1452480 (PMC11629706; doi:10.3389/fcimb.2024.1452480)
Supplement: Supplementary file 1 [file DataSheet1.zip › mendelian test/ebi-a-GCST90027696.csv_leave_one_out.pdf]

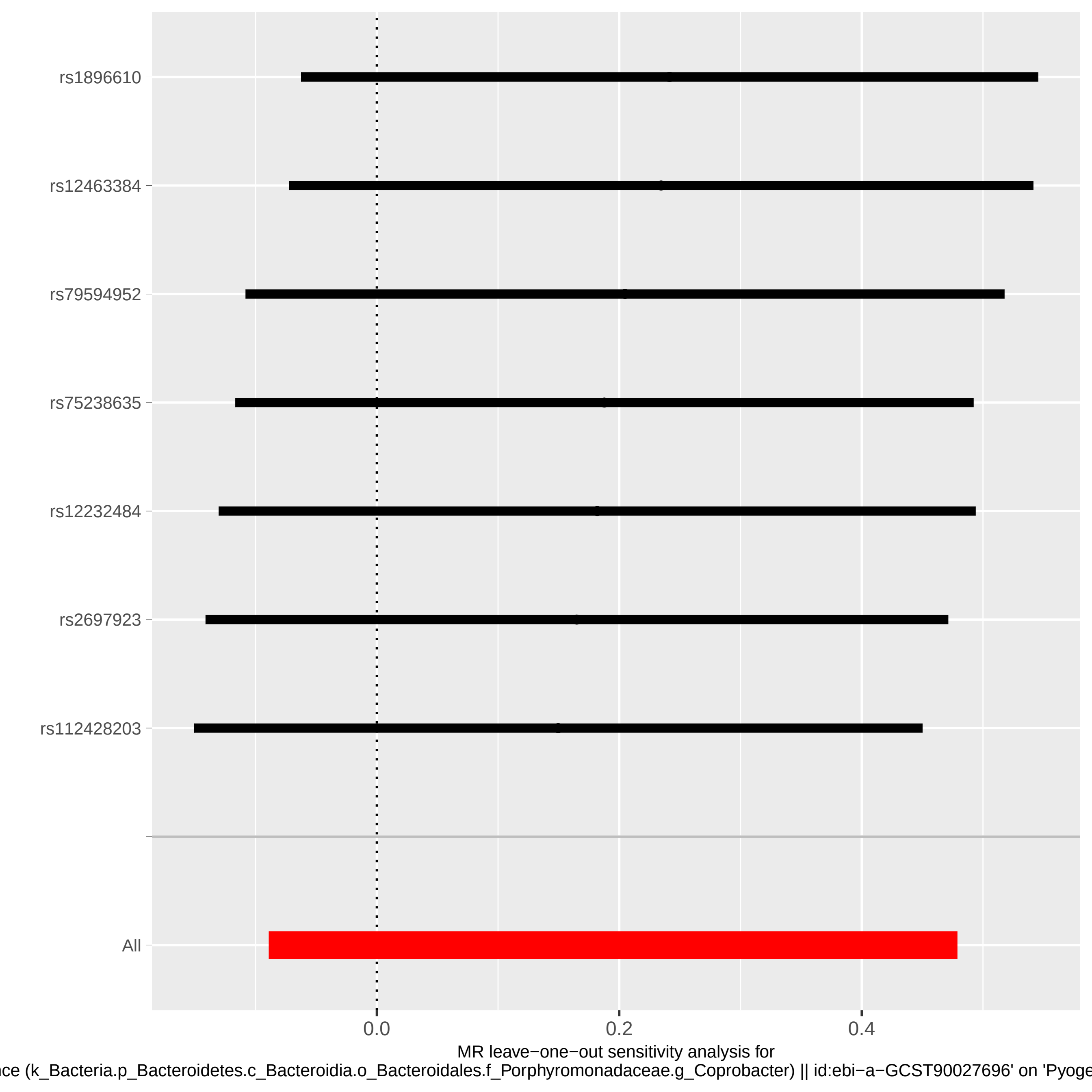
rs1896610
rs12463384
rs79594952
rs75238635
rs12232484
rs2697923
rs112428203
All
0.0
0.2
0.4
MR leave−one−out sensitivity analysis for
nce (k_Bacteria.p_Bacteroidetes.c_Bacteroidia.o_Bacteroidales.f_Porphyromonadaceae.g_Coprobacter) || id:ebi−a−GCST90027696' on 'Pyoge
